# The Importance of HHLA2 in Solid Tumors—A Review of the Literature

**DOI:** 10.3390/cells13100794

**Published:** 2024-05-07

**Authors:** Agnieszka Kula, Dominika Koszewska, Anna Kot, Miriam Dawidowicz, Sylwia Mielcarska, Dariusz Waniczek, Elżbieta Świętochowska

**Affiliations:** 1 Department of Oncological Surgery, Faculty of Medical Sciences in Zabrze, Medical University of Silesia, 41-808 Katowice, Poland; d201069@365.sum.edu.pl (M.D.); dwaniczek@sum.edu.pl (D.W.); 2 Department of Medical and Molecular Biology, Faculty of Medical Sciences in Zabrze, Medical University of Silesia, 19 Jordana, 41-800 Zabrze, Poland; s85874@365.sum.edu.pl (D.K.); s85876@365.sum.edu.pl (A.K.); d201109@365.sum.edu.pl (S.M.); eswietochowska@365.sum.edu.pl (E.Ś.)

**Keywords:** HHLA2, KIR3DL3, TMIGD2, immunotherapy, immune checkpoint, B7 family

## Abstract

Cancer immunotherapy is a rapidly developing field of medicine that aims to use the host’s immune mechanisms to inhibit and eliminate cancer cells. Antibodies targeting CTLA-4, PD-1, and its ligand PD-L1 are used in various cancer therapies. However, the most thoroughly researched pathway targeting PD-1/PD-L1 has many limitations, and multiple malignancies resist its effects. Human endogenous retrovirus-H Long repeat-associating 2 (HHLA2, known as B7H5/B7H7/B7y) is the youngest known molecule from the B7 family. HHLA2/TMIGD2/KIRD3DL3 is one of the critical pathways in modulating the immune response. Recent studies have demonstrated that HHLA2 has a double effect in modulating the immune system. The connection of HHLA2 with TMIGD2 induces T cell growth and cytokine production via an AKT-dependent signaling cascade. On the other hand, the binding of HHLA2 and KIR3DL3 leads to the inhibition of T cells and mediates tumor resistance against NK cells. This review aimed to summarize novel information about HHLA2, focusing on immunological mechanisms and clinical features of the HHLA2/KIR3DL3/TMIGD2 pathway in the context of potential strategies for malignancy treatment.

## 1. Introduction

The immune system prevents organisms from developing infections and tumors, and disturbance of its homeostasis mechanisms destroys the protective barrier against cancer development. The interactions of immune cells within the tumor microenvironment induce immunosuppression and lead to the survival and progression of tumors. Immune checkpoint proteins are surface molecules on certain immune cell populations that stimulate or inhibit immune function [1]. Numerous studies have indicated an interaction between the expression of the co-inhibitory protein and tumor immune escape. Consequently, blocking the interaction between an immune checkpoint protein and its ligands emerges as a promising therapeutic approach [2,3,4,5]. Cancer immunotherapy is a rapidly developing field of medicine that aims to use the host’s immune mechanisms to inhibit and eliminate cancer cells. This treatment enhances and stimulates humoral and cellular immunity, often leading to lower toxicity than chemotherapy or radiation [6]. There are various types of immunotherapies, such as immunostimulatory cytokines, oncolytic viruses, adoptive cell transfer, and tumor-targeting antibodies, among the spectrum of immunotherapies, monoclonal antibodies (mAbs) targeting immunosuppressive signals on cancer or immune cells, commonly recognized as immune checkpoint inhibitors (ICIs), are most present in practice today, with multiple U.S. FDA approvals across solid tumors, bring promising effects of cancer treatment [7]. Inhibiting molecules that negatively control T cell activity allows the immune system to eliminate tumors effectively. Antibodies targeting CTLA-4 (Cytotoxic T-lymphocyte associated protein-4), PD-1 (Programmed cell death protein 1), or its ligand PD-L1 (Programmed death-ligand 1), are used in various cancer therapies [8]. However the most thoroughly researched pathway with checkpoint-blocking antibodies targeting PD-1/PD-L1 has many limitations, and various malignancies resist its effect. Moreover, cancer activates other pathways to escape the immune system [9]. One of the critical pathways in inhibiting the immune response is HHLA2/receptors. In various cancers, HHLA2 is expressed higher than PD-L1. According to recent studies, there is no correlation between HHLA2 and PD-L1 in cancers, which suggests that the mechanism of the immune system inhibition of HHLA2 and PD-L1 is entirely independent [10,11,12,13]. Therefore, HHLA2/receptors may be a potential new block pathway improving cancer immunotherapy.

This review aimed to summarize novel information about HHLA2, focusing on immunological mechanisms and clinical features of the HHLA2/KIR3DL3/TMIGD2 pathway in the context of potential strategies for malignancy treatment.

## 2. B7 Family

T cells play a crucial role in anti-cancer defense; their activation, at the first step, involves the recognition of an MHC class antigen by the TCR receptor, and the second step entails the interaction of co-regulators such as proteins from the B7 family [14]. The B7 family is crucial in maintaining the balance between immune potency and preventing autoimmunity and comprises ten molecules, including well-known B7-H1 (PD-L1), B7-1 (CD80), B7-2 (CD86), B7-DC (PD-L2), B7-H2 (CD275), B7-H3 (CD276), B7-H4, B7-H5, B7-H6, and B7-H7 (HHLA2) [15].

B7 family molecules consist of membrane-anchored protein ligands that bind to the CD28 family receptors on immune cells and regulate immune responses via costimulatory or coinhibitory pathways [14]. The B7-CD28 family is phylogenetically divided into three groups, and HHLA2 is classified in group III. Research has revealed that various inhibitory checkpoints of the B7 family are elevated in tumors, leading to the spread of cancer cells by immunosuppression; thus, blocking the B7 family members could enhance anti-tumor immune responses.

## 3. HHLA2 Structure and Expression

Human endogenous retrovirus-H Long repeat-associating 2 (HHLA2, known as B7H5/B7H7/B7y) is the youngest known molecule from the B7 family, which was first reported in 1990. HHLA2 is encoded on human chromosome 3q13.13 and shows 23–33% similarity in amino acid levels with other B7 family representatives. The HHLA2 protein is built of N-terminal signal peptide, an ectodomain composed of tandem IgV-IgC-IgV domains, six potential sites for N-linked glycosylation, a transmembrane region, and a 49-aa cytoplasmic tail.

Several features distinguish HHLA2 from other molecules of the B7 family, such as three extracellular immunoglobulin domains (IgV, IgC, and IgV); the majority of members in the B7 family have an IgV and IgC domain as a standard [16,17].

Moreover, HHLA2 has orthologs only in humans and monkeys but not in mice or rats. This property generates limitations in conducting in vivo research; thus, humanized animal models are created using human cancer cell lines growing in mice to perform lab experiments. Additionally, it was discovered that receptors for HHLA2 are also present only in primates. The HHLA2/receptors pathway is necessary and critical in humans but not essential in rodents; it was possibly developed through the evolution of the immune system [17].

The expression of HHLA2 occurs primarily in carcinomas of the breast, lung, thyroid, ovary, pancreas, liver, and gastrointestinal tracts; the localization of the protein is both membranous and cytoplasmic in tumor cells. Additionally, it is limited in healthy tissues such as the kidneys, placenta, and intestines and is constitutively expressed on APC-human macrophages, activated dendritic cells, and induced B cells (after stimulation with IFN-γ) [9]. The expression rates in different tumors are shown in Table 1, whereas in Table 2, we present the influence of HHLA2 expression on the immunological landscape. Studies indicate that regulating this molecule’s expression in cancer cells differs from HHLA2 pathways in monocytes [17]. In several tumors, HHLA2 expression is higher than PD-L1, PD-L1-negative; tumors are often HHLA2-positive [18]. Yituo Xu et al. proved that in HCC, HHLA2 expression was negatively correlated with PD-L1, indicating that their expression may be differently regulated in cancer. Moreover, Haiying Cheng et al. noticed frequent HHLA2 expression in PD-L1-negative tumors in lung cancer [19,20]. These results suggest that the HHLA2 and PD-L1 pathways have different independent regulation mechanisms.

Recent studies have pointed out the critical role of macrophages in cancer development. Macrophages can be divided into the M1 fraction, which stimulates the immune system by secreting pro-inflammatory cytokines, and the M2 fraction of macrophages, which secrete anti-inflammatory factors to inhibit the immune response [21]. Tumor-associated macrophages (TAMs) act as M2 macrophages and constitute approximately 50% of stromal cells in the tumor microenvironment. They play a significant role in cancer cell survival, generating the immuno-suppressive area by the secretion of inflammatory mediators, growth factors, cytokines, and chemokines; they are also involved in angiogenesis and tumor cell metastases [19,22]. HHLA2 is a molecule that is highly expressed in TAMs. Yangzhi Qi et al. studied the correlation between TAMs and HHLA2 in glioma. They found that TAMs were significantly higher in the HHLA2 low-expression group, which indicates the crucial role of HHLA2 in tumor microenvironment (TME) organization and the process of monocytes developing into TAMs. Thus, the HHLA2/TAMs pathway could be a new target for therapies [1,20].

**Table 1:** HHLA2 expression rate, associated prognosis, and potential role of HHLA-2 in different tumors (when expression rate is not cited, the rate of high HHLA2 expression/HHLA2 upregulation is named).

Type of Tumor	Expression Rate	HHLA-2 Expression and Prognosis	Potential Mechanisms	References
colorectal cancer	83.7%	Unclear	Unclear—potential co-stimulatory and co-inhibitory effects; EMT and cell cycle regulation	[23,24,25]
gastric cancer	53.2% (high expression)	Unfavorable prognosis in the case of elevated HHLA-2 expression in tumor tissues	Unclear	[26]
renal cancer	94.57%	Unclear	EMT regulation	[12,27,28,29,30]
hepatic cancer	49.0%–67.7%	Unfavorable prognosis in the case of elevated HHLA-2 expression in tumor tissues	Inhibitory effect on immunological responses	[13,31,32,33,34,35]
gallbladder cancer	53.68%–100%	Unfavorable prognosis in the case of elevated HHLA-2 expression in tumor tissues	EMT promotion	[36,37]
pancreatic cancer	77%	More favorable prognosis in the case of HHLA-2 expression in tumor tissues	Stimulation of immunological responses	[38,39]
lung cancer	11.1% (NSCLC); 68.6% (adenocarcinoma)	Unfavorable prognosis in the case of elevated HHLA-2 expression in tumor tissues	Cell cycle and EMT regulation	[19,38,40,41]
ovarian cancer	17.19%–100%	Unfavorable prognosis in the case of elevated HHLA-2 expression in the stromal compartment	Unclear	[42,43]
breast cancer	56% (upregulation)	Unclear	Unclear	[44,45]
urothelial cancer	77.8%	UTUC—more favorable prognosis in the case of increased HHLA-2 expression in tumor cells; BUC—unfavorable prognosis in the case of elevated HHLA-2 expression	Unclear	[46,47]
thyroid cancer	100%	Unfavorable prognosis in the case of elevated HHLA-2 expression in tumor tissues	Inhibition of immunological responses	[48,49]
cervical cancer	97.4%	More favorable prognosis in the case of increased HHLA-2 expression in tumor tissues	Unclear	[50]

NSCLC—non-small cell lung carcinoma; UTUC—upper tract urothelial carcinoma; BUC—bladder urothelial carcinoma; EMT—epithelial–mesenchymal transition.

**Table 2:** Influence of HHLA2 expression on immunological landscape in tumors.

Type of Tumor	HHLA2 Expression and Immune Infiltration		References
	Increase in	Decrease in	
colorectal cancer	CD4+ resting cells, activated dendritic cells, eosinophils	CD8+ T cells, NK resting cells, M0 macrophages	[23,25]
renal cancer	CD8+ cells	-	[12]
hepatic cancer	CD4+ Foxp3+ cells (ICC), M0 macrophages, neutrophils, follicular helper T cells, memory-activated CD4+ T cells, Tregs, dendritic cells (HCC) (unclear)	CD3+, CD8+ cells (ICC), M2 macrophages, monocytes, activated mast cells, NK cells (HCC), CD4+, CD8+ cells, Tregs (HCC) (unclear)	[13,31,33,35]
gallbladder cancer	-	CD8+ cells	[37]
pancreatic cancer	CD8+ cells	-	[38]
lung cancer	M2 macrophages (adenocarcinoma)	-	[19]
ovarian cancer	CD8+ cells	-	[43]
thyroid cancer	-	CD8+ cells	[49]

“-“—data not available; HCC—hepatocellular carcinoma; ICC—intrahepatic cholangiocarcinoma.

## 4. HHLA2 Receptors—Immunosuppression and Immunostimulation

Previously, only one receptor for HHLA2 was known, which is why contradictory studies proved the co-stimulatory or co-inhibitory role of this molecule in cancer development. Discovering another receptor for HHLA2 clarified that this molecule has a dual effect on T cell activation. Depending on the receptor bound, it can stimulate or inhibit T cell proliferation, generating different effects on cancer development [11,17,18,51]. The connection of HHLA2 with the CD28 family member transmembrane and immunoglobulin domain containing 2 TMIGD2, also known as CD28H and IGPR1, induces T cell growth and cytokine production via an AKT-dependent signaling cascade [43]. TMIGD2 is a membrane glycoprotein that belongs to the immunoglobulin superfamily (IgSF), which is expressed in naive T cells and NK cells, and with the activation of T cells, the expression of TMIGD2 is gradually lost [52].

**Figure 1:**
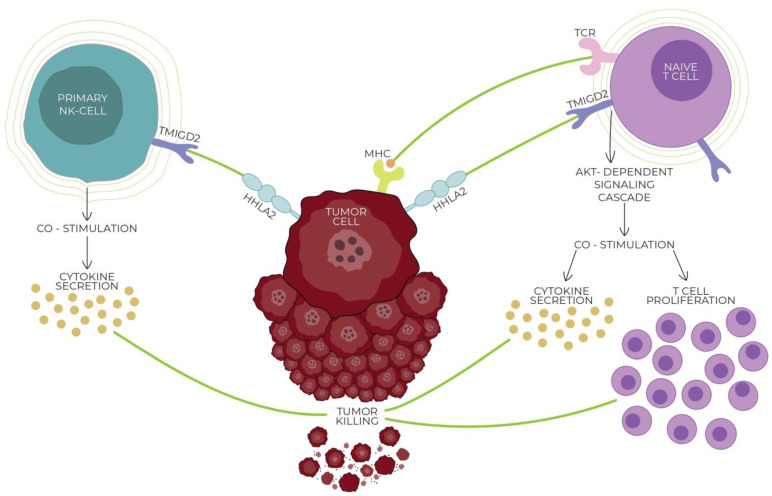
Interaction of HHLA2 with the CD28 family member transmembrane and immunoglobulin domain containing 2 (TMIGD2; also known as CD28H and IGPR1), expressed on naive T/NK cells. HHLA2/TMIGD2 is one of the critical pathways in modulating the immune response. The connection of HHLA2 with TMIGD2 induces T cell growth and proliferation and cytokine production via an AKT-dependent signaling cascade, weakening tumor cells and killing them. With the activation of T cells, the TMIGD2 receptor is gradually lost, decreasing the co-stimulatory abilities of HHLA2. Both the receptors and ligands of the HHLA2–KIR3DL3–TMIGD2 pathway are found only in mammals; this phenomenon does not occur in other members of the B7 family.

**Figure 2:**
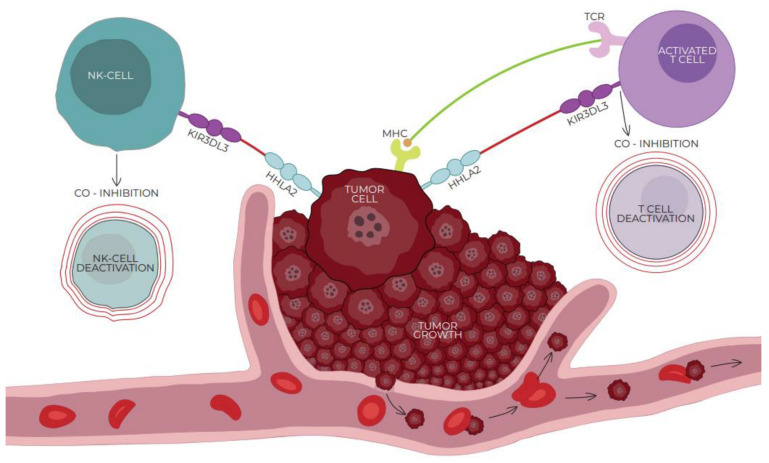
Interaction of HHLA2 and co-inhibitory receptor killer cell immunoglobulin-like receptor, three immunoglobulin domains, and long cytoplasmic tail 3 (KIR3DL3) expressed on activated T/NK cells. HHLA2/KIRD3DL3 is one of the critical pathways in modulating the immune response. The connection of HHLA2 with KIR3DL3 leads to the inhibition of T cells and mediates tumor resistance against NK cells, affecting tumor growth in an NK cell-dependent way. With the activation of T cells, the expression of KIR3DL3 increases, promoting the co-inhibitory abilities of HHLA2. Both the receptors and ligands of the HHLA2–KIR3DL3–TMIGD2 pathway are found only in mammals; this phenomenon does not occur in other members of the B7 family.

On the contrary, Wei et al. and Bhatt et al. discovered independently that an HHLA2-mediated signal through the killer cell immunoglobulin-like receptor, three immunoglobulin domains, and long cytoplasmic tail 3 (KIR3DL3) decreases T cell activation significantly and suggested that KIR3DL3 is an inhibitory checkpoint receptor [18,51]. KIR3DL3, also known as KIRC1, KIR44, and KIR3DL, is a member of the inhibitory receptor KIR family that contains an immunoreceptor tyrosine-based inhibitory motif (ITIM). KIR surface receptors possess three extracellular immunoglobin domains and only one inhibitory motif within the long cytoplasmic domain [51,53,54]. Wie et al. found that KIR3DL3 is expressed primarily in CD8+ and NK cells, binding with HHLA2, which leads to the inhibition of T cells and mediates tumor resistance against NK cells. With the activation of T cells, the TMIGD2 receptor is gradually lost while the expression of KIR3DL3 increases, promoting the co-inhibitory abilities of HHLA2 [18]. Figure 1 and Figure 2 show the interactions between HHLA2 and the co-stimulatory receptor (TMIGD2)/co-inhibitory receptor (KIR3DL3). Both the receptors and ligands of the HHLA2–KIR3DL3–TMIGD2 pathway are found only in mammals; this phenomenon does not occur in other members of the B7 family [19]. 

## 5. HHLA2 vs. Hot and Cold Tumors

Because of the infiltration of lymphocytes, cytokine secretion, molecular signatures of immune activation, and response rates to immunotherapy, tumors can be divided into hot (T cell inflamed) and cold tumors (non-T cell inflamed) [55]. Microsatellite instability (MSI) is a result of the defects in DNA mismatch repair proteins (MSH2, MLH1, PMS1, PMS2, MSH6, or MSH3) and occurs in various cancer types, such as ovarian, skin, and about 15% of gastrointestinal cancers. Microsatellite-instability-high (MSI-H) tumors correlate with a high tumor mutation burden (TMB), which is the total number of mutations identified in the tumor genome. MSI and TMB are examples of response markers to ICB therapy [56]. In bioinformatic analysis, Yang et al. demonstrated that HHLA2 expression was correlated with TMB and MSI of various cancer types, indicating that high HHLA2 expression was positively correlated with the increased expression of TMB and MSI of a cancer [57]. According to various studies, highly mutated tumors are more likely to express immune checkpoints. Tumors sensitive to immunotherapy (hot tumors) show microsatellite instability and high TMB, which are reflected in increased PD-L1, PD1, and CTL4 expression [58]. Mutations in the DNA mismatch repair (MMR) of the genes correlate positively with mutation-associated neoantigens (MANAs) in the TME, implicating high environmental immunogenicity and sensitivity to immunotherapy. TMB is directly proportional to the increased MANA secretion in the TME. A lack of defects in MMR genes (cold tumors) causes low MANA levels in the TME but not in the pro-inflammatory environment—insensitive to immunotherapy. A stimulation of MANA expression in cold tumors may sensitize them to immunotherapy [59,60]. Recently, it has been proven that MSS tumors can also respond to immunotherapy. Several trials have investigated how to turn cold tumors into hot ones, which aim to combine immunotherapy with other methods, e.g., chemotherapy, radiotherapy, tyrosine kinase inhibitors, antiangiogenetic or anti-EGFR agents, or a combination of various immune checkpoint inhibitors [60,61]. Further studies are needed to search for high-expression checkpoints in MSS tumors and identify the mechanisms of their sensitization to immunotherapy. Our recent work studied associations between HHLA2 and microsatellite instability in colorectal cancer. We observed the increased expression of HHLA2 both in MSI and MSS CRC tumors. Overexpression of this checkpoint makes it noteworthy for research on CRC immunotherapy [25].

## 6. HHLA2 and Cancer Stem Cells

Tumors are a heterogeneous population of cells, including subgroups with a high tumorogenic potential. Cancer stem cells (CSCs) are a small subpopulation of highly resistant tumor cells with characteristics of self-renewal, infinite proliferation, and potential for multi-directional differentiation. The exact origin of cancer stem cells remains unknown; the theory of their existence was first shown in breast cancer cells [62,63]. The main consequences of CSCs are recurrence after primary treatment, tumor cell heterogeneity, metastasis, minimal residual disease, and drug resistance. Conventional treatment, such as chemotherapy, is not able to eliminate the entire CSC population. CSCs can survive; they can enter a dormant state after the treatment and become a source of tumor recurrence [64]. A total cancer therapy resulting in a complete cure is often impossible because of the lack of CSC eradication. Thus, eliminating CSCs is one of the biggest therapeutic challenges in modern oncology. Min Luo et al. found that HHLA2 expression was significantly associated with poorly differentiated HCCs; this may be directly related to the promotion of CSC formation by HHLA2 [32,65]. Poorly differentiated cancer cells cannot repair degraded tissue; in such an environment, the proliferation of CSCs is promoted. Our previous study searched for HHLA2-related pathways activated in CRC by GSEA. In the analysis, we found a relationship between HHLA2 and genes critical for the cell cycle and proliferation, such as HALLMARK_E2F_TARGETS. E2Fs regulate cancer stem cells (CSCs) significantly, contributing to the proliferation, self-renewal, metastasis, and drug resistance of CSCs [25,66]. These results show that there may be a relationship between HHLA2 and cancer stem cells. However, further research is needed to evaluate the mechanisms between them.

## 7. HHLA2 in Different Malignancies

### 7.1. HHLA2 in Colorectal Cancer

In colorectal cancer, tissues exhibit HHLA2 expression in the majority of cases. In the study conducted by Zhang et al., only 16.3% of CRC tumor tissue specimens did not express HHLA2, while high expression was observed in 38.3% of the cases [67]. The intensity of HHLA2 staining was visibly stronger and revealed HHLA2 localization on the cytoplasm and membrane of cancer cells and in the stromal fibroblasts. Overall, 30 out of 63 tumor tissues (47.6%) exhibited high expression of HHLA2 (H-score > 9) [23], and the upregulation of HHLA2 in cancerous tissue was confirmed in another study conducted on 159 cancerous and noncancerous specimens [25].

Ziwen Zhu et al. conducted a study on 63 patients and confirmed that HHLA2 expression was associated with the depth of invasion and correlated negatively with the infiltration of CD8+ T cells. In our recent study, HHLA2 expression was found to be related to the immune infiltration landscape in CRCs. High HHLA2 levels corresponded to an increased fraction of CD4+ resting cells, activated dendritic cells, and eosinophils and also to a lower fraction of NK resting cells and M0 macrophages. In the HHLA2 low-expression group, lower immune scores of stromal fraction, intratumor heterogeneity, aneuploidy, and proliferation were observed, while the TGF-beta response score was increased, which suggested that high HHLA2 expression can also promote immunological responses in CRC [23,25]. Moreover, in the tumor tissue obtained from patients with CRC, high HHLA2 levels corresponded to the advanced T and N stages, and more prominent distant recurrence in the study performed on 134 CRC cases [68]. The relationship between HHLA2 expression and other clinicopathological characteristics, such as gender, age, race, smoking history, or alcohol history, was not relevant [23,25,67,68].

Increased HHLA2 expression was shown as a sign of poor prognosis and was related to a high mortality rate of the patients with CRC. There was a significant negative correlation between the overall survival of the patients and HHLA2 expression, which could be considered as an independent prognostic marker in CRC (*p* = 0.039) [23]. On the contrary, Ma et al. demonstrated that in their lncRNA signature model, high HHLA2 expression corresponded to a low risk and longer overall survival for patients with CRC, while low HHLA2 levels were observed in the high-risk patients. This was confirmed by Li et al., who also found HHLA2 expression to be decreased in a high-risk group of patients with CRC [24,69]. These results suggest that the downregulation of HHLA2 in colorectal cancer may be associated with poor prognosis.

The role of HHLA2 in CRC is unclear. It is likely to be involved in critical pathways such as epithelial–mesenchymal transition (EMT), apoptosis, proliferation, and cell cycle regulation. HHLA2 may play a limited costimulatory role by binding to TMIGD2, but it can also alleviate inflammatory responses via KIR3DL3, and these conclusions need to be confirmed in future research [25]. Because of the limited number of studies and contradictory results, it is uncertain whether HHLA2 can be considered a potential prognostic marker for colorectal cancer. Its function is multidimensional and requires further clarification.

### 7.2. HHLA2 in Gastric Cancer

The role of HHLA2 in gastric cancer is also controversial. In the earliest available study conducted by Shimonosono et al., qRT-PCR revealed significantly higher levels of HHLA2 mRNA in peripheral blood mononuclear cell (PBMC) specimens of healthy volunteers in comparison with HHLA2 mRNA copies in blood specimens of patients with gastric cancer (*p* < 0.0001). HHLA2 expression in cancer tissues showed a weak linear relationship with HHLA2 mRNA blood levels. HHLA2 in gastric cancer tissues was expressed mainly in the membrane or cytoplasm of both healthy epithelial cells and tumor cells [70]. In opposition to these results, Wei et al. uncovered that HHLA2 expression is upregulated in gastric cancer. The authors examined 408 gastric cancer and 211 normal tissue specimens and found HHLA2 to be significantly upregulated in the tumor tissues. Moreover, HHLA2 mRNA and protein levels were increased in the cancer tissue compared with the paired adjacent normal tissue. Immunohistochemistry revealed high HHLA2 expression in 53.2% of tumor tissues [26]. The patients with low HHLA2 expression presented more prominent distant metastasis, advanced tumor node metastasis (TNM) stages, and stages of the depth of tumor invasion [26,70]. However, in the study conducted by Wei et al., HHLA2 was positively related to clinicopathological parameters, such as lymph node metastasis, distant metastasis, deep tumor invasion, and advanced clinical stage, while the relationships with age, gender, tumor location, histological grade, venous invasion, and Lauren’s classification were not relevant [26]. Interestingly, Mansorunov et al. found no relationships between HHLA2 expression and metastases in gastric cancer in 101 samples. However, HHLA2 correlated with the expression of LGALS3—another immune checkpoint gene encoding Galectin-3—at stages I and II, and, additionally, correlated negatively with HER2 expression at stage IV [71]. These data may be important in the context of developing anti-tumor HHLA2 inhibitors in the future because the co-expression of other immune checkpoint genes could modify the therapeutic effects of targeting this molecule.

The patients with high HHLA2 expression presented a shorter OS (HR = 3.321 2.123–5.193 *p* < 0.001). Furthermore, elevated HHLA2 expression was identified as an independent unfavorable prognostic marker for the overall survival of the patients with gastric cancer [26]. On the contrary, Shimonosono et al. showed that the 5-year survival rate of the patients with low HHLA2 expression was lower than in the high expression group (46.5% compared with 77.6%) and the HHLA2 level was positively associated with better overall survival in univariate analysis (*p* = 0.0004). However, the study had a retrospective character, and the number of patients included was relatively low (*n* = 111), with a low number of healthy volunteers (*n* = 20) [70]. The authors suggest that the contradictions between the studies of Shimonosono et al. and Wei et al. might have been caused by a weak negative relation between HHLA2 expression in tumor tissues and HHLA2 mRNA levels in the blood [26].

The prognostic role of HHLA2 in gastric cancer remains to be elucidated. More research with larger sample sizes would be required to clarify its exact function and clinical potential in this type of cancer.

### 7.3. HHLA2 in Renal Cancer

HHLA2’s role in renal cancers has been extensively evaluated. Several studies aimed to investigate HHLA2 expression in clear cell renal cell carcinoma (ccRCC), demonstrating its significant upregulation in cancerous tissue in comparison with normal tissues [27,72,73]. Using qRT-PCR, Chen et al. showed an increased HHLA2 expression in 94.57% of tumor tissues obtained from patients with ccRCC. Immunohistochemistry revealed strong HHLA2 staining in 50% of the specimens [27,73]. According to TCGA and GEO datasets, ccRCC tissues exhibited higher HHLA2 mRNA levels than the matched normal tissues [28,72].

The tumor environment seems to play a crucial role in maintaining HHLA2 expression in ccRCC cells in vivo. It was shown that cell cultures obtained from HHLA2-positive specimens exhibited a gradual decrease in HHLA2 protein and mRNA expression in vitro and restored it after reintroducing them in vivo. The mechanism allowing for tumor cells to maintain HHLA2 expression does not depend on cytokines, hypoxia, lactic acidosis, glucose-deprived environments, or demethylation. Moreover, the regulation of HHLA2 expression differs between monocytes and kidney cancer epithelial cells. The mechanism maintaining HHLA2 expression in ccRCC cells remains to be elucidated, as it may be crucial for developing successful strategies of immunotherapy in the future [74].

HHLA2 expression correlated positively with the expression of other markers including PD-L1, PD-L2, and B7-H6, while there was a negative association between HHLA2 and B7-H3 expression. On the contrary, another study showed that HHLA2 was not correlated with the expression of other B7 or CD28 family proteins, suggesting its independence [72,74]. Interestingly, Zhou et al. found that in ccRCC tissues, HHLA2 expression was more prominent than PD-L1 both in the training and validation cohorts—the authors found 44.2% (91/206) and 41.1% (81/197) HHLA2-positive samples and 33.0% (68/206) and 32.0% (63/197) PD-L1-positive samples, respectively [12]. Moreover, HHLA2 expression has been associated with the expression of ferroptosis-related gene ChaC Glutathione Specific Gamma-Glutamylcyclotransferase 1 (CHAC1), another prognostic factor in clear cell renal cell carcinoma [75]. In ccRCC, HHLA2 expression has been shown to associate with patient clinicopathological characteristics. HHLA2-positive groups exhibited a larger maximum tumor dimension, higher lymphovascular invasion, and elevated risk of disease progression and all causes of death, while high HHLA2 expression corresponded strongly to the advanced TNM stage, higher Fuhrman grade, and necrosis, suggesting that.

HHLA2 expression in ccRCC is a sign of poor prognosis [12,27]. Several studies suggest that high HHLA2 levels correspond to a lower overall survival rate and lower progression-free survival of patients and present HHLA2 as an independent risk factor for prognostic prediction. Similarly, in the study performed by Zhou et al., the group expressing both HHLA2 and PD-L1 displayed lower PFS and OS and was related to advanced TNM grade and necrosis [12,27,28].

Lujun Chen et al. confirmed that in ccRCC progression, HHLA2 promotes the epithelial-to-mesenchymal transition (EMT) by modulating EMT markers. Knockdown of HHLA2 reduced the migration and decreased the proliferation of ccRCC cells. 

Chen et al. identified numerous long non-coding RNAs, circular RNAs, and mRNAs as deregulated in cells expressing HHLA2, suggesting them to be possible HHLA2 targets. GO and KEGG pathway analysis indicated that mRNAs deregulated in the presence of HHLA2 were related to biological processes and took part mainly in immunological pathways [27].

Genetic alterations in the context of HHLA2 expression were also analyzed by Zhang et al. According to the TCGA dataset, there were significant differences in copy number alterations between high and low HHLA2 groups, with the former presenting a higher rate of diploid normal copy, and the latter—a higher proportion of single-copy deletions. Additionally, HHLA2 expression was associated negatively with DNA methylation in five out of the seven investigated sites. Similarly, changes in DNA methylation occur in papillary renal cell carcinoma (PRCC) according to TCGA. In this case, the HHLA2 gene promoter showed that hypomethylation and HHLA2 expression exhibited a significant negative correlation with DNA methylation [76]. Consistent with the results of Chen et al., genes upregulated in the high-HHLA2 group were related to immunological responses, such as chemotaxis and cytokine production, which indicated a positive relationship between inflammatory activity and HHLA2 expression [72].

Although the correlations between HHLA2 expression and prognosis in renal cell carcinoma are unclear, several authors included it in highly reliable prognostic models for RCC.

Liu et al. built a prognostic survival model for pRCC with eight methylation-driven genes. They found that HHLA2 was the second most commonly altered gene and that the levels of HHLA2 methylation correlated positively with a risk score. Its hypermethylation and downregulation were associated with poor survival, possibly by enabling the immune escape of the cancer cells [29]. Another prognostic model with HHLA2 for ccRCC was constructed by Zhang et al. The authors used three other genes, NFE2L3, IFI16, and ZNF582. The four genes were found to undergo significant methylation and expression changes in tumor tissue compared with healthy renal tissues. In this model, HHLA2 upregulation correlated with a favorable prognosis, while a high-risk score was associated with shorter overall survival, disease-specific survival, and progression-free interval. The poor prognosis was related to alterations in inflammatory responses, the cell cycle, and adaptive immune-related processes [30]. Similarly, a high-risk score in the prognostic signature of Zhou et al. was associated with lower OS, more advanced tumor grade, and T and M stages. Additionally, in a three-gene model (CTLA4, TNFSF14, and HHLA2, with HHLA2 expression being negatively associated with the risk score) developed by Fan et al., a high-risk score was connected to the upregulation of immune responses. The enhanced responses in the high-risk group included increased activity of antigen-presenting cells and stronger type II IFN responses, as well as a more prominent infiltration of dendritic cells, CD8+ T cells, macrophages, T helper cells, follicular helper T cells, and regulatory T cells. The high-risk subgroup presented a higher TIDE score, meaning a worse response to immunotherapy. These results may also implicate the connection of the risk score—and lowered HHLA2 expression—with immunosuppressive responses [77].

In conclusion, enhanced HHLA2 expression in RCC may promote immunological responses in the tumor environment, thus contributing to a better prognosis for patients, but this relationship is unclear. Prognostic models suggest that HHLA2 downregulation is a sign of increased immunosuppressive responses, meaning that HHLA2 plays an important role in enhancing immunological activity against tumor cells in renal cancer. However, the inconsistent data implicate that the immunosuppressive properties of HHLA2 may take part in the process as well, revealing the complex nature of HHLA2’s role in RCC. Further studies in this area are of high importance as not only is HHLA2 a potential target for immunotherapy in renal cancer, but epigenetic changes related to its expression may also be valuable in the therapeutic context. The mechanism underlying the regulation of its expression may bring valuable insights into the influence of the tumor microenvironment on cancerous cells.

### 7.4. HHLA2 in Hepatic Cancer

Most studies found HHLA2 to be upregulated in liver tumors. Jing et al. found that in intrahepatic cholangiocarcinoma (ICC), HHLA2 expression is weak in peritumoral tissues, while 49.0% and 67.7% of patients in the training and validation cohorts, respectively, exhibited strong HHLA2 expression [13]. HHLA2 was also overexpressed in hepatocellular carcinoma (HCC) compared with para-carcinoma regions and healthy tissues; moreover, HHLA2 mRNA was expressed prominently in tumor tissue compared with adjacent tissue in 67 HCC cases [66]. On the other hand, in the study by Xu et al. on 205 HCC samples, immunohistochemistry revealed more intense HHLA2 staining in peri-tumoral regions compared with tumor and non-tumor tissues. This expression occurred mostly in CD68+ monocytes/macrophages, of which 71.1% were HLA-DR-positive. Importantly, HHLA2 expression in Mo/Mφs was barely detectable in normal conditions, suggesting that it could be induced along with macrophage activation in the tumor microenvironment [31,34].

Some correlations between the expression of HHLA2 and other immune checkpoints have been observed in hepatic malignancies as well. In HCC in a TCGA cohort, HHLA2 expression correlated positively with the expression of PVR, LGALS3, CD276, LGALS9, CD80, CTLA4, VSIR, LAIR1, and CD86 [35]. Certain authors also observed a negative relation between HHLA2 and PD-L1 levels, while, according to other reports, there was no association between the expression of both molecules in liver tumors. Interestingly, HHLA2 in positive HCC cells co-cultured with macrophages demonstrated higher PD-L1 expression, and this effect was not observed in HHLA2-high cells alone, suggesting that HHLA2 overexpression may enhance PD-L1 expression by promoting the polarization of M2 macrophages [31,32]. Notably, HHLA2 expression could influence the response of patients to chemotherapy: low HHLA2 levels corresponded to an elevated resistance to sorafenib or imatinib treatment. Examining HHLA2 expression in HCC could help in predicting the outcomes of immunotherapies [35]. Additionally, higher HHLA2 levels correlated with higher CEA levels in the serum of patients with ICC and with the expression of CD73—another potential immuno-therapeutic target whose expression is associated with poorer OS and immunosuppression in ICC [13,78].

The expression levels of HHLA2 were found to be correlated positively with tumor invasion of adjacent structures, poor tumor differentiation, and advanced Barcelona Clinic Liver Cancer (BCLC) classification stage in HCC [66]. Moreover, high HHLA2 expression was associated with multiple tumors, microvascular invasion, tumors with hepatic capsule invasion, and a more advanced clinical stage (*p* = 0.04). There was also a positive association between increased HHLA2 mRNA levels and a more advanced TNM stage in HCC [31,32], as well as the advanced stage in ICC. Notably, HHLA2 seems to have a crucial influence on the immunological landscape in liver malignancies. Increased HHLA2 expression correlated negatively with intratumoral CD3+ and CD8+ tumor-infiltrating lymphocyte (TIL) counts in ICC, while HHLA2-high specimens exhibited elevated CD4+ Foxp3+ scores, implying that HHLA2 could silence immunological responses during the disease [13]. It was found that the HHLA2-high subgroup of patients with HCC was characterized by elevated fractions of M0 macrophages, neutrophils, follicular helper T cells, memory-activated CD4+ T cells, Tregs, and dendritic cells, while there was a decrease in the fractions of M2 macrophages, monocytes, activated mast cells, and resting NK cells [31,35]. The average CD8+ density was also higher for HHLA2-positive than for HHLA2-negative samples [9]. At the same time, Guo et al. uncovered a negative correlation between HHLA2 expression and CD4+, CD8+, Treg, and NK cell infiltration according to the TCGA database by CIBERSORTx, and similar results were presented by Luo et al. [32,33]. Moreover, Wang et al. observed increased M2 infiltration in HHLA2-high tumors, which contradicts the results obtained by Ding et al., who proved that the proportion of M2 macrophages was lower in the case of higher HHLA2 expression. HHLA2 staining intensity was associated positively with the intensity of CD11b, a myeloid-derived suppressor cell marker [31,32], and the association was confirmed in another report. Ding et al. demonstrated that the levels of markers characteristic for exhausted CD8+ T cells were augmented in the case of high HHLA2 expression in comparison with a HHLA2-low subgroup, and their level correlated positively with the density of CD8+ T cells. Patients with higher HHLA2 expression exhibited poorer anti-tumor immunological responses, suggesting that despite more abundant immune infiltrations, immune cells might be deprived of their functions in HCC with elevated HHLA2 expression [34,35]. Despite differences in the results, the studies show that immunological responses are compromised in liver tumors in the event of elevated HHLA2 expression, meaning that HHLA2 is likely to induce immune escape in ICC and HCC.

In ICC, elevated HHLA2 expression was associated with worse OS. Notably, the authors pointed out that HHLA2 could be a better prognostic marker than PD-L1 in ICC [13]. In HCC, high HHLA2 expression also corresponded to a poorer prognosis. Moreover, Ding et al. found that HHLA2 is the most important immune checkpoint protein in terms of survival prediction in HCC. Higher HHLA2 expression corresponded to a shorter OS and a higher probability of recurrence in the TCGA cohort [31,32,33,34,35]. Additionally, high simultaneous expression of HHLA2 and PD-L1 was correlated to worse survival than in the case of low expression of either of these molecules. On the contrary, in another study, patients positive for HHLA2 had a lower mortality rate than patients negative for HHLA2 after 56 months (12.7% and 37.5%, respectively), suggesting HHLA2 expression to be a sign of good prognosis in HCC. In the same study, HHLA2 expression related to a better disease-specific survival (DSS), while the best prognosis was associated with HHLA2-positive cases with high CD8+ density [9,34]. Interestingly, Luo et al. discovered that the increased HHLA2 expression was associated with a shorter OS only in patients with low and intermediate TIL density, but for patients with high TIL density, no such correlation was observed. These results suggest that HHLA2 may play an immunosuppressive role in the presence of a low number of immune cells in the tumor microenvironment [32].

The molecular function of HHLA2 in HCC and ICC has been studied extensively. An analysis of the profiles of 371 patients with HCC in the TCGA database revealed 19,922 differently expressed genes that related to the expression of HHLA2. Most closely related DEGs (differently expressed genes) were involved in immunological responses [66]. Differently expressed genes in patients with high HHLA2 included neural cell adhesion molecule 1 (NCAM1), CD3, CD4+, and Granzyme B (GZMB), which presented lower expression, as well as T cell Ig and ITIM domain (TIGIT), CD19, and CTLA4, which were upregulated, suggesting enhanced immunosuppressive responses in the event of increased HHLA2 expression [34]. In another study, a HHLA2-high subgroup of patients with HCC presented the highest enrichment ratio in genes involved in pathways related to the cell cycle. Tumor tissue also had a lower rate of HHLA2 methylation. Interestingly, methyltransferase genes were downregulated in tumors and the adjacent tissue regardless of the HHLA2 expression level, suggesting that methylation may be the factor influencing HHLA2 expression in liver cancer [35].

HHLA2 knockdown (KD) in HepG2 cells increased cell adhesion and apoptosis but decreased cell invasion, migration, and differentiation 3 days after transfection with the lentivirus, as well as reduced the number of cells progressing to the phase G2/M of the cell cycle, compared with the controls [66]. On the other hand, HHLA2 overexpression in vitro caused higher Hep3B and MHCC97 cell proliferation. HHLA2 silencing in mice resulted in a slightly decreased incidence of HCC (6/10 compared with 8/10 in WT mice), nodular metastases, and CD31 (a vascular marker) expression. IHC showed, as well, lower PD-L1 expression and TAM levels in tumor tissues of HHLA2 KD in mice. When transplanted to the liver of the mice, Hep3B cells with silenced HHLA2 exhibited a lowered growth rate and lung metastasis rate [31,33]. This means that HHLA2 may promote HCC progression, being a potential immunotherapeutic target, especially for patients with advanced stages of the disease. In conclusion, the interactions between HHLA2 and immune cells in the tumor microenvironment are a multidimensional issue, which hopefully will be addressed in future reports in more detail.

### 7.5. HHLA2 in Gallbladder Cancer

Studies show that HHLA2 could play a significant role in gallbladder cancer (GBC) as well. Zhang et al. found that among 89 examined patients, all cases exhibited HHLA2 staining [36]. In another study, high HHLA2 expression was observed in 53.68% of samples in the training group and 60.19% of cases in the testing group. HHLA2 presented a significant co-expression rate with other B7 family members. B7-H3 and HHLA2 were expressed together in 43.16% and 48.54% of samples, while B7-H4 was co-expressed with HHLA2 in 37.89% and 43.69% of cases in the training and the testing groups, respectively. These co-expression patterns could influence the effects of HHLA2 targeting in immunotherapies, therefore it is important to be mindful of this data [36,37]. Moreover, HHLA2 expression in GBC was more prominent than PD-L1 expression, suggesting that it could be a better therapeutic target in gallbladder cancer than PD-L1 [79].

High HHLA2 expression was correlated with tumor progression, tumor invasion, more advanced Nevin stage, AJCC stage, and regional lymph node metastasis. Notably, according to other authors, higher Nevin and TNM stages were associated with the high expression of HHLA2, B7-H3, and B7-H4 in both the training and testing groups. Elevated HHLA2 expression was also correlated with a lower density of CD8+ TILs in the training group (*p* = 0.001), but there was no relationship between HHLA2 expression and Foxp3^+^ TILs [36,37].

The patients with low HHLA2 expression exhibited longer OS (*p* = 0.01), while high expression of B7-H3, B7-H4, and HHLA2 corresponded to worse OS and CRS (cancer-related survival). Additionally, HHLA2 was an independent risk factor for poor OS (*p* < 0.001) and CRS (*p* = 0.007) in GBC in the training group, but not in the testing group—possibly because of the correlations between HHLA2 and other immune checkpoint genes [36]. Lv et al. suggested that other B7 family members should be considered in prognostic models involving HHLA2. Indeed, a subgroup of patients showing strong simultaneous expression of B7-H3, B7-H4, and HHLA2 (B7-high grade subgroup) exhibited shorter OS and CRS than other subgroups. The authors demonstrated that the B7-high grade group had a more advanced Nevin stage, TNM stage, and poorer survival rates in terms of OS and CRS than the B7-low grade group (*p* < 0.001) [37].

Zhang et al. also examined the possible molecular mechanism of HHLA2 expression in GBC, measuring the expression of α-SMA, vimentin, N-cadherin, and Col-I—EMT markers—in HHLA2-overexpressing GBC-SD cells. The levels of the mentioned markers were elevated, except for the E-cadherin expression. These results indicate that HHLA2 may be a factor promoting EMT in GBC [36,37,80].

Altogether, HHLA2 is a promising target for immunotherapy in gallbladder cancer. It should be noted, however, that the expression of other B7 family members must be taken into account when considering therapeutic strategies, as their co-expression is likely to influence the effects of such therapies. Still, more studies with a higher number of samples should be encouraged to elucidate HHLA2’s significance in gallbladder cancer.

### 7.6. HHLA2 in Pancreatic Cancer

HHLA2 was found to be upregulated in pancreatic cancer. Yan et al. examined pancreatic ductal adenocarcinoma (PDAC) specimens and found that HHLA2 was expressed in 71 out of 92 cases. Healthy cells of peritumoral tissues (duct cells, islets, and acinar cells) did not exhibit HHLA2 expression, but in pancreatic intraepithelial neoplasia (PanIN) lesions of the peritumoral area (detected in 20 out of 91 cases), HHLA2 immunohistochemical staining was mostly positive. Moreover, some inflamed ductal cells showed HHLA2 expression [39,81]. In the independent intraductal papillary mucinous neoplasm (IPMN) cohort, 70.73% of cases expressed HHLA2, but this expression was not markedly related to IPMN grade. The HHLA2 expression rate was especially high for intestinal and pancreaticobiliary types. The adjacent healthy tissue was generally negative for HHLA2. Similar results were obtained in a study of 122 patients with pancreatic cancer and 72 patients with ampullary cancer of the pancreaticobiliary subtype. In total, 77% of tumors were positive for HHLA2, and HHLA2 mRNA was detected in all 23 examined cancer tissues. Notably, mRNA levels did not associate with HHLA2 protein levels, indicating that HHLA2 may be expressed mainly in the tumor microenvironment and not predominantly in cancer cells [38]. High HHLA2 expression was also observed in pancreatic neuroendocrine tumors (PNETs). On the other hand, Zhu et al. found no significant difference between HHLA2 expression in cancer and healthy tissues, although the sample amount in their study was relatively low (*n* = 63). However, the authors detected HHLA2 expression mainly in tumor-associated macrophages (TAMs) and stromal cells [82,83].

Importantly, HHLA2 expression in cancer cells correlated with the expression of another B7 family molecule—B7-H3 (*p* < 0.0001)—and the association between HHLA2 and B7-H4 expression was negative. Similarly, in CK−CD8−CD68− area cells and CD68+ TAMs, the expression of HHLA2 correlated positively with B7-H3 levels [83]. Moreover, Boor et al. suggested a negative relationship between CA-19.9 and HHLA2 [38]. Knowledge about the co-expressed immune checkpoint molecules in PC is of high importance in the context of developing successful HHLA2-based immunotherapeutic strategies in the future. Potentially, future studies will shed more light on the issue.

In a study of 92 samples, Yan et al. attempted to investigate the correlations between HHLA2 expression status and clinicopathological factors (age, gender, AJCC and TNM staging, and more), but none of them was significant [39], which was similar to the results obtained by Zhu et al. [83]. On the other hand, Boor et al. demonstrated that HHLA2 expression related positively to infiltrating CD8+ cells, suggesting that HHLA2 could promote immunological activity in pancreatic cancer [38]. Additionally, the intensity of CD68+ TAM infiltration was elevated with increasing HHLA2 expression in both tumor and CK−CD8−CD68− area cells (*p* < 0.0001). Interestingly, there was a marked decrease in tumor-infiltrating CD4+ and CD8+ T cells in PNETs with high HHLA2 and B7x expression. The study showed that HHLA2 protein and mRNA levels were elevated in primary tumor tissues, which resulted in metastasis in comparison with primary tumors without metastases (*p* < 0.001). There was also a positive association between HHLA2 expression rate and tumor size, and HHLA2 expression correlated with a higher grade and the incidence of nodal and distant spread of PNETs. This means that HHLA2 may play an important role in the progression and metastasis of PNET [82,83].

HHLA2 expression seems to be a sign of good prognosis in PDAC. The HHLA2-positive group had a significantly higher survival rate compared with the HHLA2-negative group (*p*= 0.0015), and HHLA2 expression was correlated positively with the long-term survival rate (*p* = 0.0141) [39]. Moreover, patients positive for HHLA2 exhibited delayed cancer recurrence in pancreatic cancer. HHLA2 expression was also demonstrated to be an independent predictor of cancer-specific survival [38]. Notably, Zhu et al. confirmed that high HHLA2 expression corresponded to better OS (*p* = 0.08), but interestingly, the opposite results were obtained for patients with CD68+ TAMs—in this case, high HHLA2 levels associated with poorer OS (*p* = 0.01). At the same time, the overall survival of the group of patients with HHLA2 expression in TAMs marked as B7-H3^low^HHLA2^low^ was markedly better than patients marked as B7-H3^high^HHLA2^high^, and similarly, patients marked as B7-H4^low^HHLA2^high^ had poorer survival than patients marked as B7-H4^low^HHLA2^low^ (*p* < 0.01). Low HHLA2 expression in tumor cells corresponded to and elevated death risk for patients, but increased HHLA2 expression in CK−CD8−CD68− area cells was also related to a higher risk of death (*p* = 0.01). These results indicate that HHLA2 has a prognostic value in PC. Moreover, HHLA2 expression plays different roles in immunological cells and the tumor microenvironment [83]. HHLA2 in cancer cells may stimulate immune response in pancreatic cancer, which is a sign of a good prognosis but plays the opposite role in immune cells. Understanding this mechanism requires more data, and future studies should focus on the differentiation between how HHLA2 expression in tumor cells and HHLA2 expression in other cells of the tumor microenvironment can influence the course of the disease. It is worth mentioning that high HHLA2 expression in PC corresponded to shorter OS (*p* = 0.007), according to the TCGA database, which highlights the fact that the significance of HHLA2 expression in PC remains to be clarified [81].

### 7.7. HHLA2 in Oral and Esophageal Cancer

A few authors tackled the issue of HHLA2 expression in oral (OSCC) and esophageal squamous cell carcinoma (ESCC). Xiao et al. found that OSCC tissue exhibited stronger IHC staining for HHLA2 than normal mucosa tissue, while TMIGD2 was poorly expressed in OSCC and showed a stronger expression in normal mucosa tissue samples. Neither HHLA2 nor TMIGD2 expression levels were significantly correlated with pathology grade, tumor size, or lymph node status. Moreover, there was a strong positive correlation between HHLA2 and TIM3, LAG3, B7H3, B7H4, and VISTA expression (*p* < 0.0001), while TMIGD2 was associated with higher TIM3, LAG3, and B7H3 expressions. Considering the associations between the expression of HHLA2 and other immune checkpoint molecules, OSCC patients could benefit from combined immune checkpoint blockade therapy. The authors also suggest that HHLA2 could play an immunosuppressive role in OSCC. High HHLA2 expression corresponded to shorter OS, and similar results were obtained for high expression of TMIGD2 (*p* = 0.0314 and *p* = 0.0081, respectively) [84].

Additionally, using data from 119 patients with ESCC from the GSE53623 dataset as a training cohort and 60 patients with ESCC from the GSE53622 dataset as a validation cohort, Zhang et al. constructed a two-gene prognostic model based on the B7-CD28 family. The selected genes were HHLA2 and ICOSLG; HHLA2 expression was negatively related to the risk score. The high-risk group had a shorter OS (*p* < 0.0001) and RFS (*p* = 0.0382) in comparison with low-risk patients, and the risk score was an independent prognostic factor for ESCC in a multivariate analysis. The authors also demonstrated that the high-risk score correlated with more abundant Treg cells and fibroblasts, suggesting that silenced immunological response and low HHLA2 expression may be related to poor prognosis in ESCC. On the other hand, the high-risk score correlated positively with IgG, interferon, lymphocyte-specific protein-tyrosine kinase (LCK), hematopoietic cell kinase (HCK), MHC-I, MHC-II, and signal transducer and activator of transcription 1 (STAT1), implying higher activation of macrophages and T cell signaling [85]. More studies are needed to uncover the prognostic and therapeutic potential of HHLA2 in oral or esophageal malignancies.

### 7.8. HHLA2 in Lung Cancer

Numerous studies have shown that HHLA2 is upregulated in non-small cell lung carcinoma (NSCLC) including squamous cell carcinoma, lung adenocarcinoma (LUAD), and pulmonary sarcomatoid carcinoma (PSC) [19,20,86,87,88]. The authors found that HHLA2 was present in the majority of specimens assessed, while the most normal lung tissue specimens were HHLA2-negative. According to Chen et al., in normal tissue, HHLA2 is not detectable in endothelial cells, alveolar cells of type I and II, or smooth muscles in blood vessels, but it appears occasionally in bronchial epithelial cells [40]. HHLA2 was also more prominent in adenocarcinoma (68.6% of analyzed cells) than in squamous cells (35.4%) and large-cell (11.1%) NSCLC. The increased expression of HHLA2 in lung adenocarcinoma was detectable mainly in the tumor cell cytoplasm and membrane [40,86]. Similarly, Long et al. reported that HHLA2 is mainly expressed in the cell membrane and cytoplasm of the immune cells (77.0% of patients displayed high expression) and LUAD cells (14.9% of patients) with more prominence than in non-cancerous cells. The authors also detected HHLA2 expression in the bronchial epithelium and smooth muscle cells [89].

There were significant correlations observed between the H-score for HHLA2 and genomic alterations including KRAS and *MET*ex14 mutations. The former presented a lower H-score for HHLA2 in the epithelial component, while the latter was correlated to a higher IHC score for HHLA2 in both components. On the other hand, the HGF/*MET*ex14 pathway is likely not to influence HHLA2 expression but to increase PD-L1 expression [88]. Additionally, HHLA2 expression was found to be elevated in lung cancer with *EGFR* mutation—one of the most common mutations in lung cancer [41,86,90].

HHLA2 expression in lung cancer is associated significantly with tumor characteristics and clinicopathological features. HHLA2 expression correlated positively with a higher TIL score [86,90]. As reported by Chen et al., hHHLA2-Ig inhibited TCR-mediated proliferation in 43% of CD4+ and 26% of CD8+ T cells in comparison with control hIgG. It also markedly decreased the secretion of eight cytokines including IFNγ, TNFα, IL13, IL9, IL10, IL17A, IL4, and IL22, indicating the suppressive role of HHLA2 on TCR-dependent T cell proliferation and cytokine production in NSCLC [20]. Additionally, the TIMER database uncovered a correlation between higher M2 macrophage infiltration and increasing HHLA2 expression in lung adenocarcinoma. Silencing HHLA2 caused the expression of M2 macrophage markers to drop, suggesting an inhibitory effect on M2 polarization, possibly because of IL-10 downregulation [19]. In another study, 74.2% of 62 lung carcinoma samples expressed HHLA2, and HHLA2 expression correlated with metastases. Only 44.4% of patients with no metastases exhibited HHLA2 expression in the tumor tissue in comparison with 83% of patients with metastases, and patients with stage IV of the disease exhibited the highest percentage of HHLA2-positive cases. The levels of HHLA2 expression and the mentioned characteristics were not statistically significant [41]. HHLA2 expression was not reported to be related to the smoking history of patients but correlated positively with age in elderly patients with LUAD [89]. On the contrary, Wang et al. found no significant association between HHLA2 expression and clinicopathological factors in PSC [88].

Chen et al. demonstrated that a less abundant expression of the molecule is associated with longer disease-free survival in lung adenocarcinoma and suggested HHLA2 as a promising target, especially in lung cancer with altered EFGR [40]. Similar results were obtained by Farrag et al., although the overall survival of patients positive for HHLA2 was approximately 50% shorter than the OS of patients negative for HHLA2. On the other hand, the progression-free survival was markedly lowered (*p* = 0.01). The results suggest that HHLA2 may be valuable for prognostic purposes. However, the study was conducted on a small number of biopsy specimens (*n* = 62) and has a retrospective character, meaning that other factors influencing the results could have been ignored [41].

Importantly, HHLA2 silencing led to a smaller tumor size in vivo, reduced the viability of A549 and H1299 cells, and decreased Ki67 levels. Flow cytometry revealed that HHLA2 targeting causes cell cycle blockage at the G0/G1 checkpoint, while immunoblotting showed decreased CyclinD1/E1 and CDK2/4/6 levels with P27 upregulation upon HHLA2 silencing. Moreover, HHLA2 knockdown led to markedly lowered healing rates in A549/H1299 cells, decreased migration and invasion, reduced N-Cadherin, Vimentin, MMP2, and MMP9 levels, and resulted in E-Cadherin upregulation. It also resulted in lowered EGFR, MEK, and ERK1/2 phosphorylation, possibly inhibiting EGFR/MAPK/ERK signaling [19]. These results show that targeting HHLA2 could be a beneficial therapeutic strategy, and the research on this topic is of high importance.

Interestingly, in the study conducted by Cheng et al., HHLA2 was co-expressed with both PD-L1 and B7H4 in 13% of cases, and triple-positive specimens were present mainly in stage III lung cancers. In this group, 26% of cases were triple-positive, while the percentage was significantly lower for stages I and II (12%). Importantly, 78% of PD-L1-negative cases appeared to express HHLA2, B7H4, or both [20]. Wang et al. demonstrated that HHLA2 was expressed in all epithelial components that did not exhibit PD-L1 expression and in 66.7% of sarcomatoid PD-L1-negative components in PSC specimens [88]. HHLA2 expression also correlated positively with the expression of another B7 family protein in immune cells—VISTA [89]. This indicates that HHLA2 could be an appropriate alternative for targeting PD-L1 in lung cancer. The authors suggest that targeting new immune checkpoints such as HHLA2 is beneficial for patients suffering from NSCLC, especially those who exhibit resistance to the present immunotherapy. However, the sample size in the majority of the studies available is relatively low, and the studies are mostly retrospective. More reports concerning the potential therapeutic role of HHLA2 in lung cancer are needed to establish its role and clinical significance.

### 7.9. HHLA2 in Ovarian Cancer

The role of HHLA2 in ovarian cancer (OC) is not clear, and the number of studies on the topic is limited. Examining 160 ovarian cancer tissues and 119 epithelial ovarian cancer (EOC) cases, Fu et al. found that all samples exhibited HHLA2 expression [42]. IHC revealed HHLA2 staining in tumor, stromal, and immune cells, and the median score of HHLA2 expression in the tumor compartment was significantly higher than in the stromal compartment (31.5% compared with 7.35%), indicating stronger HHLA2 expression in the tumors (*p* < 0.0001) [42]. On the contrary, in a study conducted by Xu et al., most cancer tissues were HHLA2-negative—only 11 out of 64 samples presented HHLA2 expression [43]. On the other hand, normal ovarian epithelium presented a significantly higher HHLA2 expression rate, with 43.75% positive samples (*p* = 0.041). These results were confirmed with the use of the Oncomine dataset, which showed that cancer tissue has a significantly lower HHLA2 expression compared with normal ovarian tissue (*p* = 0.004) [43]. Therefore, the upregulation of HHLA2 in OC remains uncertain.

HHLA2 expression was not correlated significantly with PD-L1 nor B7x expression in ovarian cancer [43]. However, it was induced in neutrophils after MUC16 treatment, meaning that MUC16 (CA125)—a marker protein for OC that is likely associated with immunosuppressive responses and neutrophil-related inflammation—can influence HHLA2 expression [91].

There was a positive correlation between HHLA2 expression in the tumor compartment and the age of the patients in a study conducted on 119 EOC cases, but other studies failed to confirm this association. Interestingly, the high expression of HHLA2 in the stromal compartment was associated with more prominent lymph node metastasis (*p* = 0.002), distance metastasis (*p* = 0.049), and more advanced FIGO Stage (*p* = 0.045) [42]. On the other hand, higher HHLA2 expression was associated with a higher differentiation of ovarian cancer, but only 64 OC samples were included in this study. Moreover, the samples exhibiting HHLA2 expression presented higher CD8+ T cell density than the HHLA2-negative group, suggesting enhanced immune responses and less aggressive phenotypes [43].

Notably, in the study conducted by Fu et al., a higher level of HHLA2 in the stromal compartment corresponded to a lower survival rate of patients with EOC; however, there was no significant correlation between HHLA2 expression in the tumor compartment and OS [42]. This indicates that more studies are needed to establish the prognostic potential of HHLA2 in patients suffering from OC.

Importantly, HHLA2-overexpressing cells had reduced viability compared with controls. Moreover, they presented lower EdU incorporation in the EdU assay, indicating that cell proliferation and tumor growth were decreased in the event of HHLA2 overexpression [43]. This would mean that HHLA2-overexpressing tumors have less aggressive phenotypes. In opposition to these results, Fu et al. uncovered that HHLA2 knockdown resulted in a decrease in cell vitality, migration, and invasion in OC-derived cell lines, which means that HHLA2-deprived ovarian cancer cells exhibited less malignant behavior [92]. The tumors derived from such cells had also lower mass and smaller size. Additionally, Western blotting revealed increased expression of phospho-IKKβ and phospho-RelA—genes related to NF-κB signaling—after HHLA2 silencing. The expression of CA9a hypoxia-inducible factor-1 (HIF-1) target gene, carbonic anhydrase IX (CA9), which is a marker of tumor and poor survival—was also reduced at mRNA and protein levels. Notably, in cells with silenced HHLA2 and overexpressed CA9, viability, invasion, and migration were not lowered, and similar results were obtained for OC cells overexpressing CA9 [93]. This indicates that HHLA2 may influence the aggressive phenotype of OC cells by regulating CA9 expression. HHLA2 silencing or CA9 overexpression elevated the expression of p-IKKβ and p-RelA. The authors suggested that HHLA2 silencing upregulates the NF-κB signaling pathway, thus decreasing CA9 expression and inhibiting OC progression [92]. The research to date presents contradictory conclusions regarding HHLA2’s role in ovarian cancer. It would be beneficial to investigate HHLA2 expression and function in OC in a larger group of patients.

### 7.10. HHLA2 in Cervical Cancer

To the best of our knowledge, a study conducted by Byun et al. was the only one aiming to characterize the role of HHLA2 in cervical malignancies. In a group of 76 patients suffering from cervical adenocarcinoma (AC), HHLA2 expression was observed in 97.4% of tumor samples, and 81.6% of them exhibited high HHLA2 expression. Meanwhile, 47.4% of patients expressed PD-L1, and 54.9% of samples with strong HHLA2 expression co-expressed PD-L1. On the other hand, 85.7% of HHLA2-low tumors did not present PD-L1 expression. Overall, HHLA2 was associated positively with PD-L1 levels (*p =* 0.006). Additionally, HHLA2 expression was negatively correlated with LN metastasis (*p =* 0.011)—an independent prognostic factor for DFS and OS in AC. The HHLA2-high subgroup was also characterized by a significantly lower recurrence rate and higher disease-free survival (DFS) rate with borderline significance (*p* = 0.057), but the correlation with OS was not significant. However, overall survival was longer in patients expressing PD-L1 (*p* = 0.032). These results indicate that elevated HHLA2 and PD-L1 expression may be a sign of good prognosis in cervical adenocarcinoma. Studies including more patients are needed to establish the role and clinical potential of HHLA2 in AC and its possible co-expression patterns with other immune checkpoint molecules [50].

### 7.11. HHLA2 and Breast Cancer

There are not many reports tackling the issue of HHLA2 expression in breast cancer. Janakiram et al. found that B7-H7 expression was upregulated in 56% of patients with early-stage triple-negative breast cancer (TNBC). The authors suggested that an increase in gene copy number is one mechanism of HHLA2 upregulation in TNBC. In patients with TNBC, high HHLA2 expression was associated with advanced disease stage at diagnosis and lymph node involvement, but was unrelated to tumor size and age [44].

Another study showed that a model, based on differently expressed genes related to ferroptosis may predict the long-term prognosis of patients with high accuracy. It has been shown that immune-related pathways take part in the process of ferroptosis and HHLA2, which was found to be differently expressed in breast cancer and could be involved in ferroptosis during the disease [45]. It is unclear whether HHLA2 has prognostic or therapeutic value in breast cancer. Future works may shed more light on the issue.

### 7.12. HHLA2 in Urothelial Cancer

There are also reports concerning HHLA2 function in urothelial carcinoma. Lin et al. showed that HHLA2 is upregulated in bladder urothelial carcinoma (BUC) tissues compared with healthy bladder tissue. The authors detected HHLA2-positive staining in 77.8% of tumor specimens, while only 22.2% of healthy regions expressed HHLA2. Moreover, HHLA2 levels were elevated in the urine of patients with cancer compared with healthy controls [46]. There was a significant positive correlation between high HHLA2 expression and clinicopathological characteristics including tumor size, multiplicity, higher clinical stage, high grade, and lymph node metastasis. In upper tract urothelial carcinoma (UTUC), elevated expression of HHLA2 in tumor cells was associated with lower histological grade, lower NLR, and negative lymphovascular invasion, while higher HHLA2 expression in stromal cells correlated with a higher histological grade. The expression of stromal HHLA2 also correlated positively with stromal fibroblast activation protein (FAP) levels. FAP is another sign of the advanced stage and poor PFS in UTUC, and both markers could play a role in the progression of UTUC.

Interestingly, the increased HHLA2 expression in tumor cells was related to longer CSS (*p* < 0.01) and PFS (*p* = 0.02), but the protein expression in stromal cells was not significantly associated with these parameters. Such results suggest that tumor and stromal HHLA2 may perform different functions in the tumor microenvironment in UTUC [47]. In bladder urothelial carcinoma, HHLA2 expression is an independent risk factor for lymph node metastases. The elevated HHLA2 expression corresponded to poorer 5-year RFS (*p* < 0.001) and significantly shorter OS (*p* < 0.001). Importantly, HHLA2 had a diagnostic value for BUC—it demonstrated 82% sensitivity and 87% specificity in differentiating healthy controls among patients with BUC (AUC = 0.84). More research on the role of HHLA2 in urothelial carcinoma would be beneficial [46]. Uncovering the diverse functions of HHLA2 expression in different tumor microenvironment cells would be another exciting target for future studies.

### 7.13. HHLA2 in Thyroid Cancer

Niu et al. showed that in patients with papillary thyroid carcinoma (PTC), HHLA2 mRNA expression levels were significantly higher in PTC tissues than in normal tissues, and significant HHLA2 upregulation was uncovered in PTC cells compared with normal cells (*p* < 0.001) [48]. Similarly, the expression of HHLA2 was observed in medullary thyroid carcinoma (MTC) tissues but not in normal tissues. Moreover, high HHLA2 levels in the patients with PTC significantly correlated with more advanced AJCC and TNM stages and with more prominent lymph node metastasis, while HHLA2 expression was not significantly associated with other clinical features such as gender, age, tumor size, multifocality, depth of invasion, or extrathyroidal invasion. Similar results were obtained for MTC, where HHLA2 expression correlated positively with the advanced AJCC stage. Moreover, in the patients with MTC, CD8+ TIL levels were significantly lower in the high-HHLA2 expression group compared with the low expression group, suggesting the immunosuppressive function of HHLA2. HHLA2 overexpression also contributed to the accelerated progression of PTC cells, while the opposite effect on PTC cell progression was exerted by HHLA2 knockdown [49]. These results indicate that HHLA2 can promote tumorigenesis in thyroid cancer and can be considered an immunotherapeutic target for the disease.

High HHLA2 expression corresponded to a lower survival rate in PTC, and HHLA2 was found to be an independent prognostic factor for the patients suffering from the disease (*p* = 0.034). In MTC, it was shown that the tumor-free survival rate was lower in the patients with high HHLA2 expression compared with the patients with low HHLA2 expression. High HHLA2 expression was an independent prognostic factor for disease-free survival in the patients with MTC [48].

More research is needed to establish the prognostic and therapeutic role of HHLA2 in patients with thyroid malignancies.

## 8. Future Directions

In the prospect of potential immunotherapies targeting HHLA2, it is important to establish its co-expression patterns with other immune checkpoint genes in various types of cancer, as they could affect the effects of such treatment. Such co-expression has been observed in several malignancies, but the results of previous studies are not often in agreement [28,32,35,72,83]. Notably, in conditions such as ccRCC and gallbladder cancer, HHLA2 seems to be a better target for immunotherapy than PD-L1, which is already in use in cancer therapies [94,95]. Moreover, the HHLA2 pathway is independent of PD1/PD-L1 and, therefore, seems to be a promising target for immunotherapies in cancers resistant to any PD-L1 therapies.

Considering the dual role of HHLA2, which is an effect of its interaction with two receptors, it is possible to unblock the immune system and stimulate it, enhancing the therapeutic effect. Depending on the receptor interaction, HHLA2 has immunostimulatory or immunoinhibitory functions. Taking into account the positive function of the TMIGD2 receptor, a desirable phenomenon in therapy would be to block the interaction of HHLA2 with KIR3DL3 only. Bhatt et al. first generated monoclonal antibodies against the HHLA2/KIR3DL3 pathway that blocked KIR3DL3 inhibitory activity while preserving the TMIGD2 immune-stimulatory effects of HHLA2 [11]. A limiting factor for the studies on the HHLA2/KIR3DL3/TMIGD2 pathway is their lack of expression in rodents, significantly restricting the possibilities of standardizing the research. There are many therapeutic strategies to overcome immunotherapy resistance [17]. An interesting example of creating innovative therapeutic methods is microsatellite stable tumors—MSS in colorectal cancer. Various studies have focused on turning cold tumors into hot tumors. Several combinations have been performed to find a way to stimulate the response of MSS tumors to immunotherapy, such as immunotherapy with chemotherapy, radiation, antiangiogenetic or anti-EGFR agents, target therapy, and tyrosine kinase inhibitors, and a combination of various immune checkpoint inhibitors. Promising results are gained through a combination of second-generation CTLA4-inhibitor botensilimab plus the PD-1 inhibitor balstilimab or pembrolizumab and the anti-LAG3 favezelimab [60]. In a study on regorafenib with nivolumab, their combination allowed for synergistic enhancement to overcome the resistance to immunotherapy [96].

In July 2023, a multicenter Phase I clinical trial began using NPX887, the first-in-class monoclonal antibody targeting KIRD3LD3 to reactivate exhausted T and NK cells in HHLA2 + solid tumors that inhibit the KIR3Dl3 receptor for HHLA2. The tested treatment is intended for recurrent or metastatic solid tumors such as non-small cell lung carcinoma (NSCLC), renal cell carcinoma (RCC), colorectal carcinoma (CRC), triple-negative breast carcinoma, and more https://classic.clinicaltrials.gov/ct2/show/NCT06240728 (accessed on 1 February 2024).

## 9. Conclusions

HHLA2/TMIGD2/KIRD3DL3 is one of the critical pathways in modulating the immune response. The connection of HHLA2 with TMIGD2 induces T cell growth and cytokine production via an AKT-dependent signaling cascade. On the other hand, the binding of HHLA2 and KIR3DL3 leads to the inhibition of T cells and mediates tumor resistance against NK cells. With the activation of T cells, the TMIGD2 receptor is gradually lost while the expression of KIR3DL3 increases, promoting the co-inhibitory abilities of HHLA2. There are various cancers in which HHLA2 is expressed more frequently than PD-L1 [11,51,86]. According to many researchers, HHLA2 seems to be a promising target for immunotherapies in the cancers resistant to any PD-L1 therapies [45,89,95]. Considering the dual role of HHLA2, which is an effect of its interaction with two receptors, it is possible to unblock the immune system and stimulate it, enhancing the therapeutic effect. The role of HHLA2 in various malignancies remains controversial. In colorectal cancer, HHLA2 is expressed more prominently in the tumor than in healthy tissues, and similar tendencies can be observed in clear cell renal cell carcinoma, hepatic malignancies, gallbladder cancer, oral squamous cell carcinoma, lung cancer, bladder urothelial carcinoma, and thyroid cancer [13,23,25,28,36,46,48,49,66,67,72,84]. The issue is more complicated in the cases of gastric, pancreatic, and ovarian cancer, where studies present contradictory results regarding HHLA2 expression [26,42,43,81]. The associations with clinicopathological factors are also not clear and differ between tumor types. There are reports showing correlations between higher HHLA2 expression and more advanced T and N stages, as well as more prominent distant recurrence in colorectal cancer and a more advanced TNM stage [31,46,68]. However, in other malignancies, including gastric cancer and pancreatic cancer, authors present opposing data regarding such associations [26,38,39,70]. This is probably due to the low number of studies. Moreover, HHLA2 is involved in numerous signaling pathways in tumorigenesis, including cell cycle regulation, apoptosis, proliferation, and the epithelial–mesenchymal transition [25,36]. Targeting HHLA2 could be beneficial in cancer treatment because its silencing has been shown to improve clinicopathological conditions such as survival, decreased tumor size, cancer cell invasion, migration, and proliferation. The prognosis prediction value of HHLA2 for various cancers remains unclear. Several studies indicated unfavorable clinical outcomes associated with the overexpression of HHLA2. However, multiple reports have identified HHLA2 as a protective factor [19,33,35,36,77]. Altogether, the number of studies tackling the issue of the role and expression of HHLA2 in different types of malignancies is poor, which results in contradictory conclusions. HHLA2 gives hope for novel immunotherapeutic strategies in various tumors, but, firstly, it requires a more detailed examination. Future reports could give valuable insight into the topic and should be encouraged.
